# Estrogen Replacement Therapy in Ovariectomized Rats: Complementary Roles of ER and GPR30 in Alleviating Depressive-like Behavior

**DOI:** 10.3390/cimb48050519

**Published:** 2026-05-16

**Authors:** Siyi He, Zhongyu Ren, Lan Wu, Yinping Xie, Limin Sun, Ling Xiao, Gaohua Wang

**Affiliations:** 1 Department of Psychiatry, Renmin Hospital of Wuhan University, Wuhan 430060, China; htt2636885@163.com (S.H.); zhongyu0202@whu.edu.cn (Z.R.); wulan8@whu.edu.cn (L.W.); yinpingxie@whu.edu.cn (Y.X.); rm004016@whu.edu.cn (L.S.); 2 Institute of Neuropsychiatry, Renmin Hospital of Wuhan University, Wuhan 430060, China

**Keywords:** major depressive disorder, estrogen, GPR30, neuroinflammation, short-term stress

## Abstract

Women are twice as likely to suffer from major depressive disorder (MDD). The underlying mechanism between estrogen and depression is still unknown. We used ovariectomized rats to simulate menopausal status and established a depression model of chronic and acute stress. The therapeutic effects of estrogen were systematically studied through behavioral testing, Western blotting, ELISA, LC-MS, and cell experiments. In chronic stress, OVX rats showed depressive-like behaviors, and elevated hippocampal ER, BDNF, IL-1β/IL-18, and body weight. ERT reduced depression-like behavior by 64% to 76% in the behavioral test. ERT also reversed the molecules without affecting GPR30. In acute stress, ERT reduced depression-like behavior by 20% to 58% in the behavioral test. OVX decreased ER, BDNF, P2X7, IL-1β/IL-18, spine density, and microglia and increased the expression of GPR30. ERT reversed all the above. ERT normalized metabolic abnormalities caused by CUMS. Our study demonstrates that estrogen deficiency contributes to the onset and progression of depression in a rat model of menopause-like estrogen deficiency. Estrogen replacement therapy appears to alleviate depressive-like behaviors by reducing brain inflammation and supporting the brain’s adaptive capacities through ER. Furthermore, the dual function positions GPR30 as a promising potential target for future treatments of menopausal depression, and GPR30 regulates neuroinflammation and neuroplasticity through the NLRP3/P2X7/IL-1β pathway.

## 1. Introduction

As societal burdens escalate, depression has emerged as a major global health concern [1] associated with disability [2] and suicide [3]. It is noteworthy that the incidence of depression is twice as high in women as in men [4]. This phenomenon is mainly influenced by estrogen levels. The perimenopausal period is a natural physiological stage characterized by a gradual decline in ovarian function. During this transition, the incidence of depression increases by 2 to 5-fold within a single year [5]. However, the mechanism by which declining estrogen levels contribute to perimenopausal depression remains unclear.

Previous research has shown that the risk of developing depression in women is related to women’s sensitivity to stress. Accumulated trauma exposure has a significant impact on depression, anxiety, and somatic symptoms in women, and specific events may have a greater impact [6]. Short-term stress can significantly alter women’s cortisol and inflammatory response patterns. Female patients with depression often have dysfunction of the HPA (hypothalamic–pituitary–adrenal, HPA) axis, which may be further exacerbated by acute stress [7]. Both animal and human research have shown that women exhibit more significant HPA axis responses under stressors such as confinement or new environments [7,8]. Also, acute restraint stress can lead to changes in neuroplasticity of the NAcC (nucleus accumbens core, NAcC) and IC (insular cortex, IC), in female rats [9]. Little research has been conducted to investigate the relationship between estrogen and short-term stress.

Estrogen regulates cell function through two main mediators: the nuclear receptor family, including ERα and ERβ, and membrane receptors, including G protein-coupled estrogen receptor (GPER, also known as GPR30) [10]. The traditional estrogen receptor family is thought to be a main mediator in long-term neuroinflammation and neural plasticity [11]. These receptors exert significant effects on the pathogenesis of depression. ER could activate the PI3K/AKT/GSK3β (phosphatidylinositol 3-kinase/protein kinaseB/Glycogen Synthase Kinase 3β, PI3K/AKT/GSK3β) signaling pathway, upregulate TPH2 (tryptophan hydroxylase 2, TPH2), and promote 5-HT (5-hydroxytryptamine, 5-HT) synthesis. Collectively, these mechanisms may protect against depression [12]. Moreover, the discovery of GPR30 has significantly advanced our understanding of estrogen’s rapid, non-genomic signaling pathways.

GPR30 is widely expressed in the peripheral regions of the central nervous system, such as the hippocampus, prefrontal cortex, and hypothalamus, which are closely related to emotional and cognitive regulation [13]. GPR30 can inhibit the expression of endoplasmic reticulum stress-related proteins and neuronal apoptosis in the hippocampus, thereby improving the dysfunction caused by I/R (ischemia/reperfusion, I/R) injury [14]. Interestingly, GPR30 is widely distributed along the HPA axis [15]. Deficiency of GPR30 in the rat resulted in a significantly lower basal serum corticosterone level, but in response to acute restraint stress, GPR30-deficient rats showed a higher ACTH (adrenocorticotropic hormone, ACTH) release level, especially evident in female rats [16]. Yet, little research has been conducted to investigate estrogen-mediated signaling in short-term acute stress.

Plppr4 (Plasticity Related Gene-1, PRG-1) is a member of the Lipid Phosphate Phosphatase Related Protein 4 (LPP) family. This protein plays an important role in synaptic plasticity, affecting synaptic function by regulating palmitoylation of synaptic proteins. Research has found that palmitoylation of Plppr4 is crucial for synaptic activity-induced insertion of AMPARs into the postsynaptic membrane, thereby participating in the regulation of learning, memory, and synaptic plasticity [17]. In terms of its role in depression, abnormal expression of Plppr4 may be associated with dysfunction of depression-related neural circuits.

The P2X7 receptor is an ATP-gated cation channel belonging to the purinergic receptor family, which plays an important role in neuroinflammation, immune regulation, and cell signaling. After being activated by extracellular ATP, this receptor can trigger downstream inflammatory responses, including the release of pro-inflammatory cytokines, the generation of reactive oxygen species, and cell death [18]. In the nervous system, P2X7 receptors are mainly distributed in microglia, astrocytes, and neurons. Overactivation of this receptor promotes the activation of the NLRP3 inflammasome, leading to the release of pro-inflammatory cytokines such as IL-1 β, which in turn triggers neuroinflammation.

Given that GPR30 could mediate hippocampus neuroinflammation and other stress-related metabolism [19]. We hypothesize that estrogen deficiency induces depressive-like behavior through neuroinflammation and impaired neuronal plasticity. Estrogen replacement treatment reversed these effects via ER under chronic stress and GPR30 under acute stress. We designed a series of experiments: first, a CUMS (classical chronic unpredictable mild stress, CUMS) animal model was utilized to assess the dosage response in CUMS. We administered three different concentrations of estradiol to the ovariectomized rats and recorded the depression-like behavior of the rats. Subsequently, a short-term stress animal model was established to investigate the effect of GPR30 inhibition on the development of depression- and anxiety-like behaviors. In vitro, siRNA and GPR30 agonist G1 were used to silence or excite the GPR30 receptor in the SH-SY5Y cell line. In summary, we investigated the physiological changes and metabolic characteristics of ovariectomized rats under acute or chronic stress, as well as the mechanisms of different estrogen receptors under chronic and acute stress.

## 2. Materials and Methods

### 2.1. Subjects

#### 2.1.1. Animals

Female 60-day-old SD rats were obtained from the Laboratory Animal Center of Wuhan University, China. They were housed in polypropylene cages (55 × 40 × 20 cm) and had free access to food (supplied by China Wuhan Spring Dragon Experimental Animal Feed Co., Ltd., Wuhan, China, Food Production License Code: SCXK2025-0006, Production Standard: GB 14924.3-2010 [20], Hygiene Standard: GB/T 14924.2-2001 [21]) and water in glass bottles, except during behavioral testing. All rats were housed in a pathogen-free condition at 25 °C and 45% humidity with a photoperiod of 12 h light and 12 h dark. All animal procedures were conducted in accordance with the regulations of the National Science and Technology Committee of China and approved by the Ethics Committee of Wuhan University People’s Hospital. (IACUC Issue No. WDRM20200704).

#### 2.1.2. Cell Culture

Human neuroblastoma cells (SH-SY5Y) were purchased from Zhongsheng Aobang Biotechnology Co., Ltd. (Beijing, China). SH-SY5Y cells were cultured in a sterile constant-temperature cell incubator with a culture environment of 37 °C and 5% CO_2_. The culture medium formula consisted of 84% high-glucose DMEM + 15% fetal bovine serum + 1% penicillin/streptomycin.

### 2.2. Experiment Design

#### 2.2.1. Ovariectomy (OVX)

To eliminate the primary source of endogenous estrogen, bilateral ovariectomy (OVX) was performed. After a 14-day acclimation period, female rats were randomly assigned to either a sham-operated control group (Sham) or an OVX group. All animals were fasted for 12 h prior to surgery. Anesthesia was induced and maintained with isoflurane (induction: 3–4% in 100% O_2_; maintenance: 1.5–2% in 60% O_2_). In the OVX group, both ovaries were exposed through two dorsal incisions, and the ovaries were removed. Pedicles were ligated using chromic catgut sutures. Then the incisions were closed in layers. For the sham group, the same surgical procedure was performed except that only the same volume of fat was removed. Immediately after surgery, all rats received an intramuscular injection of cephalosporin (30 mg/kg). Injections (estradiol or vehicle) were administered in the morning (08:00–10:00) to align with circadian hormone secretion patterns.

After 21 days of recovery, the rats (*n* = 5, respectively) were randomly divided into five groups. All five groups underwent modified stressors; three of these groups received estradiol subcutaneous injections at different dosages: 30 μg/kg, 60 μg/kg, 120 μg/kg. The sham group and control group received the same volume of sesame oil.

In the following experiment, the rats (*n* = 9, respectively) were randomly divided into three groups: sham group, OVX group, and OVX + 30 μg/kg E2 group. The control group received the same volume of sesame oil. All three groups underwent short-term stress.

#### 2.2.2. Chronic Unpredictable Mild Stress, CUMS

The CUMS procedure was used to induce a depressive-like state, following established protocols with minor modifications [22,23]. Over a period of 28 consecutive days, rats were exposed daily to one or two stressors selected randomly from the following list: (a) 24 h of food deprivation; (b) 24 h of water deprivation; (c) 24 h of cage tilting at 45°; (d) 24 h of wet bedding (200 mL of water added per cage); (e) 24 h of reversed light/dark cycle; (f) 2 h of restraint in a well-ventilated plastic cylinder; (g) 5 min of tail clip (1 cm from the base of the tail); (h) 5 min of forced swimming in cold water (4 °C); (i) 5 min of forced swimming in warm water (45 °C). To maintain unpredictability, the same stressor was not applied on two consecutive days, and the sequence was randomized weekly.

#### 2.2.3. Short-Term Stress

All rats underwent unpredictable mild stress. The stressors were applied randomly and included the following mild stressors: (1) food deprivation for 24 h, (2) water deprivation for 24 h, (3) cage tilting (45°) for 24 h, (4) wet bedding for 24 h, (5) reversal of light-dark cycle for 24 h, (6) restraint for 2 h, (7) tail clip for 5 min, (8) forced swimming in cold water (4 °C) for 5 min, and (9) forced swimming in warm water (45 °C) for 5 min. The above procedure lasted for 10 days.

#### 2.2.4. Open Field Test, OFT

Before the experiment, the rats were moved from the feeding room to the behavioral testing room to adapt for at least 30–60 min to reduce transport stress. The open field test was conducted in a closed, dimly lit room (50 lx) in the afternoon (13:00–16:00). The experimental site was divided into nine areas; each rat was placed in the center of the field, allowing for free exploration. Firstly, the rats were acclimated in the open area for 1 min, followed by a tracking system (EthoVision XT 11.5) for 4 min to record the total distance traveled, average speed, number of activities, and duration of stay in the central area. During the experiment, the experimenters left the room or remained still to avoid disturbing the animals.

#### 2.2.5. Forced Swim Test, FST

The test used a transparent cylindrical container (with a diameter of about 28 cm and a height of about 40 cm) filled with water at a depth of about 30 cm, and the water temperature was maintained at 25 ± 1 °C. During the experiment, rats were placed in water and swam for 6 min. After 2 min of adoption, their behavior was recorded for the next 4 min. During the testing period, the indoor environment should maintain soft and uniform lighting to avoid additional interference.

#### 2.2.6. Sucrose Preference Test, SPT

Before the experiment, all rats were housed separately, and two water bottles containing a 1% sucrose solution were placed in each cage to allow the animals to adapt to a single cage environment. During this period, the positions of the two bottles were exchanged every 6 h to eliminate the influence of position preference on the experimental results. Then replace one bottle of sucrose with purified water and continue to switch positions every 6 h in the next 24 h. Subsequently, the rats were subjected to a 24 h water deprivation treatment. During the test, pre-weighed bottles were provided at the same time, one containing 1% sucrose solution and the other containing purified water. The positions of the two bottles should be exchanged every 6 h. The bottles were weighed after 24 h to record the liquid consumption.

Sucrose preference (%) = (sucrose water intake/(sucrose water intake + pure water intake)) × 100.

#### 2.2.7. Blood Collection, Brain Tissue Collection and Storage of Rats

After the behavioral tests, the rats were immediately deeply anesthetized with isoflurane. The rats were sacrificed for blood and brain tissue. Body weight was obtained during this procedure. The blood was collected by cardiac puncture and was then centrifuged (15 min, 3000 rpm) to obtain serum. The brain tissue was frozen in liquid nitrogen and then transferred to −80 °C freezer for storage and subsequent protein extraction; the serum was for subsequent ELISA.

#### 2.2.8. Enzyme-Linked Immunosorbent Assay, ELISA

As we previously described [22], serum estrogen concentrations were determined using an ELISA assay kit (Finetest, Wuhan, China). According to the manufacturer’s guidelines. In short, 50 μL of the diluted plasma samples or diluted standard was dispensed into each well along with the biotin-labeled antibody and was incubated at 37 °C. Following incubation with HRP-conjugated streptavidin and TMB substrate solution, the enzymatic reaction was terminated by adding sulfuric acid. The absorbance at 450 nm was then measured using a microplate reader.

#### 2.2.9. Immunofluorescence

The immunofluorescence experiments were performed as previously described [24]. The antibodies used in this study included an anti-Iba1 antibody (1:200, Huabio, Hangzhou, China) and an anti-PSD95 antibody (1:200, Cell Signaling Technology, Danvers, MA, USA). The nuclei were stained with DAPI (Sigma-Aldrich, Beijing, China), and images were captured on Leica-DMi8 (Leica, Tokyo, Japan).

#### 2.2.10. Western Blot Analysis

As we previously described [22], total hippocampus protein and cell protein were isolated using RIPA buffer (HIGH) (G2002, Servicebio, Wuhan, China). The concentration of protein was determined using the BCA Protein Colorimetric Assay Kit (Epizyme Biomedical Technology Co., Ltd., Shanghai, China) according to the manufacturer’s instructions. Then, 20 μg of protein was separated by 10% SDS-PAGE and transferred to polyvinylidene difluoride membranes (pore size: 0.45 μm, Merck Millipore, County Cork, Ireland). The membranes were then blocked for 15 min at room temperature and incubated with the primary antibody overnight at 4 °C. After five washes with 0.1% TBST, the membranes were incubated with the secondary antibody for 1 h at room temperature. Visualization was performed using the ultrasensitive ECL reagent kit (Epizyme Biomedical Technology Co., Ltd., Shanghai, China). Signal intensity was quantified using Image Lab software (version 5.2; Bio-Rad Laboratories, Hercules, CA, USA). Antibodies: Plppr4 (1:2000, Huabio, China), ER (1:1000, WanleiBio, Shenyang, China), GPR30 (1:1000, Huabio, China), BDNF (1:1000, Huabio, China), P2X7 (1:2000, Proteintech, Wuhan, China), IL-18 (1:500, Proteintech, China), IL-1β (1:1000, Abmart, Shanghai, China), cl-caspase1 (1:200, Zenbio, Chengdu, China), NLRP3 (1:1000, Huabio, China), HRP-GAPDH (1:50,000, Abclonal, Wuhan, China), and HRP-actin (1:100,000, Proteintech, China).

#### 2.2.11. Metabolic Analysis

Rats’ sera were harvested according to the manufacturer’s instructions, and the sample extracts were then analyzed using an LC-ESI-MS/MS system (UPLC: Shim-pack UFLC SHIMADZU CBM A system; MS: QTRAP System) by Metware (Wuhan, China). The raw data of the mass spectrometer were converted into mzML format by ProteoWizard (Palo Alto, CA, USA), and peak extraction, alignment, and retention time correction were performed using the XCMS program (v4.9.2). Filter the peaks with a missing rate > 50% in each group of samples and fill the blank values with KNN + 1/5 minimum value (fill the blank values > 50% with 1/5 minimum value and fill the blank values < 50% with KNN). Use the SVR method to correct the peak area. The peaks after calibration and screening were identified for metabolites by searching the laboratory’s self-built database, integrating public databases, and predicting databases. Finally, extract substances with a comprehensive identification score of 0.5 or above and a QC sample CV value less than 0.3, and then merge the positive and negative modes.

#### 2.2.12. Cell Treatment

Cells were seeded onto a six-well plate at 70% polymerization degree. Then the cells were divided into three groups. Group N was not subjected to special treatment, while the LPS modeling group was treated with 1 μg/mL LPS for 24 h. Group G1 was co-cultured with 1 μg/mL LPS and 3 μg/mL G1 for 24 h. After modeling, the cells were collected for subsequent treatment.

#### 2.2.13. Cell Transfection

SH-SY5Y cells were transfected with small interfering RNA (siRNA) targeting GPR30 or a negative control siRNA (GenePharma, Shanghai, China) using Lipofectamine 2000 (Invitrogen, Carlsbad, CA, USA) according to the manufacturer’s protocol. The siRNA sequence for GPR30 was sense 5′-CUGCAGGUCAACAUGUACATT-3′, and antisense 5′-UGUACAUGUUGACCUGCAGTT-3′. Transfection efficiency was assessed 48 h post-transfection by Western blotting.

### 2.3. Data Analysis

Statistical analyses were performed with the SPSS software (SPSS Standard version 24.0, SPSS, Chicago, IL, USA). The Shapiro–Wilk normality test and Levene’s test were applied to evaluate variance distribution and homogeneity, respectively. The statistical analysis among groups was performed using one-way ANOVA followed by Tukey’s post hoc test; when variances were unequal, Brown–Forsythe and Welch corrections were used. A *p*-value < 0.05 (two-tailed) was considered statistically significant. “ns” indicates no statistically significant difference, while * *p* < 0.05, ** *p* < 0.01, and *** *p* < 0.001.

## 3. Results

### 3.1. Dose–Response Effects of E2 in OVX Rats

**Figure 1:**
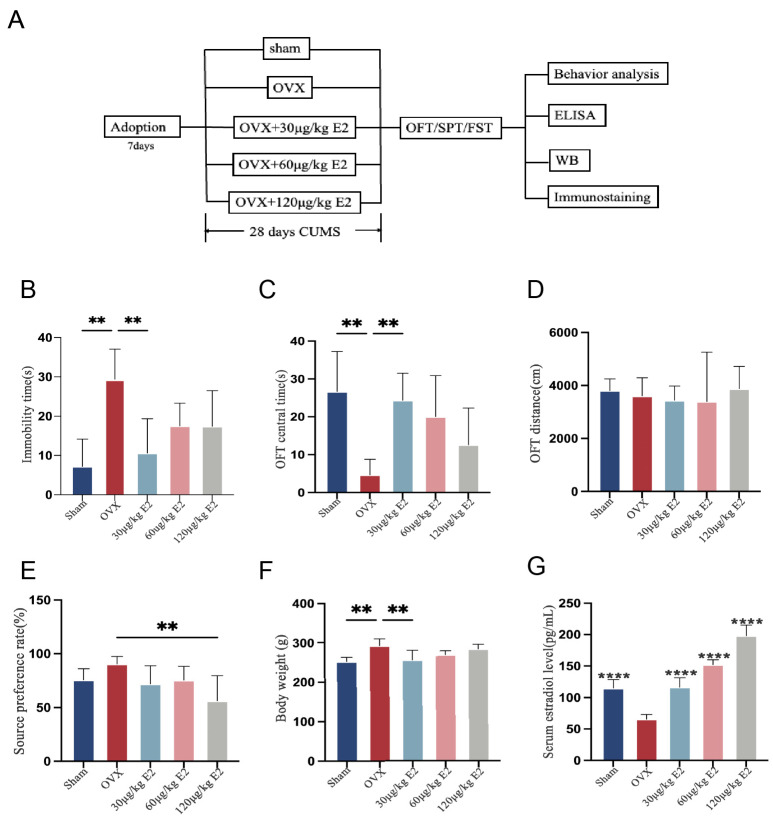
Dose-dependent effects of E2 in OVX rats. (**A**) Schematic diagram of animal experiment workflow. (**B**) The immobility time of all groups of rats in the forced swim test. The distance (**C**) and time spent in the center quadrant (**D**) are the distances of the rats in the open field. (**E**) The sucrose preference index was measured using a sugar preference test. (**F**) The body weight was measured before the rats were sacrificed. (**G**) Serum 17β-Estradiol levels in all groups of rats. Bars represent mean ± SEM. Statistical analysis was performed using one-way ANOVA with Tukey’s multiple comparisons test. ** *p* < 0.01, **** *p* < 0.0001 vs. OVX group.

After ovariectomy, rats received E2 treatment at different dosages and underwent the CUMS procedure. The detailed animal procedure is shown in Figure 1A. OVX rats with depressive-like behaviors showed improvement in the FST immobility time, when 60 μg/kg and 120 μg/kg rats did not show such a significant improvement (Figure 1B). No difference was observed in the OFT distance test among these groups, but the OVX group showed less OFT central time, which was reversed by E2 supplement. It was also recorded in experiments that OFT central time decreased as E2 dosage increased (Figure 1C,D). Interestingly, unlike other anxiety- and depressive-like behaviors, OVX rats showed a higher sucrose preference index than other groups, and we observed a decrease in 120 μg/kg OVX rats. (Figure 1E) We also measured rats’ serum E2 level; the results were as follows: the sham rats had a serum E2 concentration with a mean value of 122.352 pg/mL. The OVX rats showed the lowest E2 concentration with a mean value of 68.4 pg/mL. It is worth mentioning that the 30 μg/kg E2 group had a mean serum E2 level of 128.094 pg/mL, which is closest to that of sham rats. (Figure 1F) Also, we observed a significant weight increase in the OVX group, and supplementation with E2 could reverse this. (Figure 1G) As 30 μg/kg E2 showed significant antidepressant-like and anxiolytic-like effects, we used 30 μg/kg in the following experiments.

### 3.2. ERT (Estrogen Replacement Treatment, ERT) Alleviates Neuroinflammation and Nerve Injury Induced by CUMS via ER

**Figure 2:**
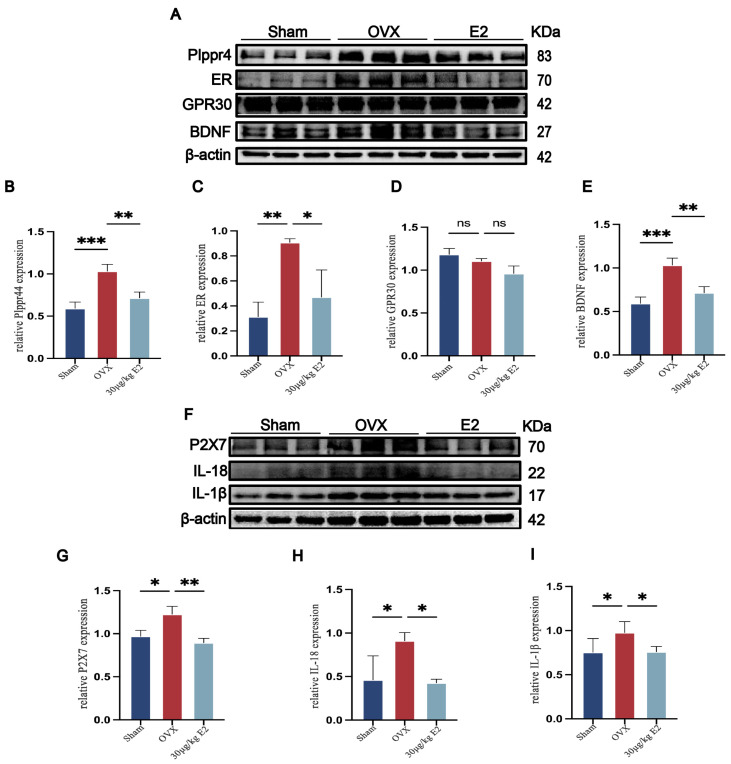
ERT alleviates neuroinflammation and nerve injury induced by CUMS via the ER. (**A**) Representative Western blots of Plppr4, ER, GPR30, and BDNF in the hippocampus. (**B**–**E**) Analysis of hippocampal Plppr4, ER, GPR30, and BDNF expression in all groups. (**F**) Representative Western blots of P2X7, IL-1β, and IL-18 in the hippocampus. (**G**–**I**) Analysis of hippocampal P2X7, IL-1β, and IL-18 expression in all groups. Data are presented as mean ± SEM. Statistical analysis was performed using one-way ANOVA with Tukey’s multiple comparisons test. * *p* < 0.05, ** *p* < 0.01, *** *p* < 0.001, ns: no significant difference vs. OVX group.

We then further explored the role of E2 (estradiol, E2) in reducing rats’ depression-like behavior. We first evaluated the expression of ER and GPR30. The OVX group showed higher expression of ER in the hippocampus (Figure 2A,C), while GPR30 expression in the hippocampus showed no significant difference in all groups. (Figure 2A,D). We then evaluated the expression of Plppr4 (phospholipid phosphatase-related 4, Plppr4) (Figure 2A,B) and BDNF (brain-derived neurotrophic factor, BDNF) (Figure 2A,E). The results showed that BDNF and Plppr4 increased in OVX rats, suggesting that ER could boost nerve protection when the rats were in a low E2 situation. Subsequently, we measured the serum IL-1β level; all samples showed low IL-1β (interleukin-1β, IL-1β), indicating that OVX did not lead to systemic inflammation after undergoing the CUMS procedure (Figure 2F). Then we examined the expression of P2X7R (Figure 2F,G), IL-1β (Figure 2F,H), and IL-18 (Figure 2F,I), all of which were significantly elevated in the hippocampus of OVX rats, and E2 supplement could relieve neuroinflammation. All the above evidence suggested that during long-term stress, E2 alleviates neuroinflammation and promotes nerve protection, and GPR30 may not be clearly involved.

### 3.3. ERT Alleviated Depressive-like Behaviors Induced by Acute Stress in Rats

**Figure 3:**
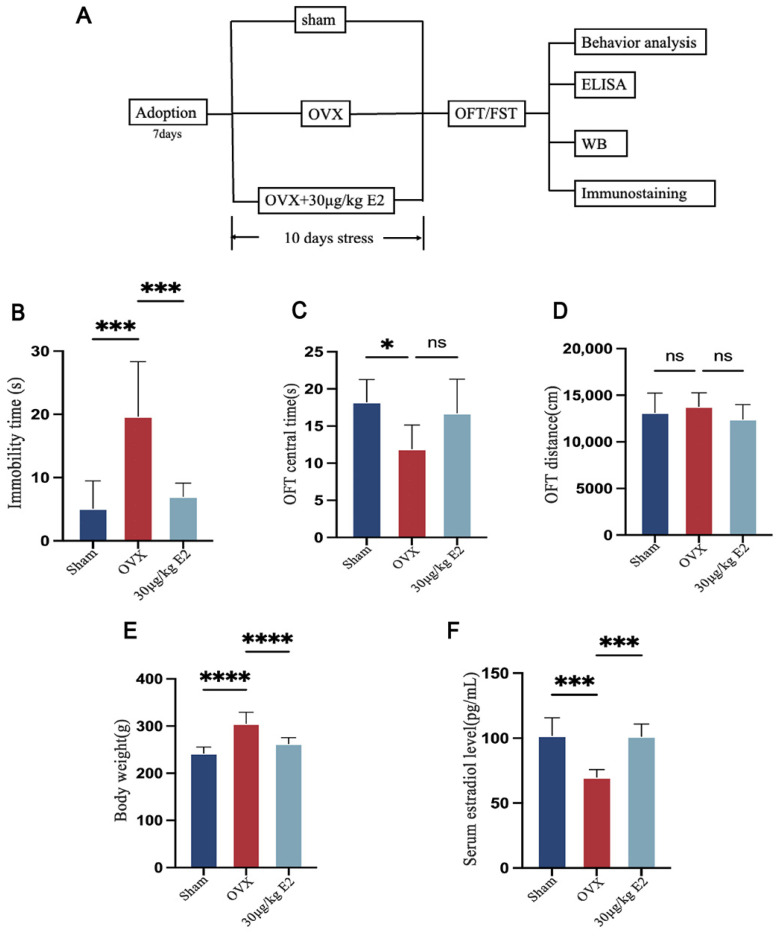
ERT alleviated depressive-like behaviors induced by acute stress in rats. (**A**) Schematic diagram of animal experiment workflow. (**B**) The immobility time of all groups of rats in the forced swim test. (**C**) The time spent in the center quadrant of the rats in the open field. (**D**) The total distance rats moved in 4 min. (**E**) The body weight was measured before the rats were sacrificed. (**F**) Serum 17β-Estradiol levels in all groups of rats. Statistical analysis was performed using one-way ANOVA with Tukey’s multiple comparisons test. * *p* < 0.05, *** *p* < 0.001, **** *p* < 0.0001, ns: no significant difference vs. the OVX group.

To further investigate the E2 antidepressant-like effect, we designed a short-term animal stress model. After ovariectomy, rats received E2 treatment at different dosages, and then the rats underwent a short-term stress procedure. Detailed animal procedure is shown in Figure 3A. Because significant body weight changes were observed in the CUMS-OVX rats, which could indicate altered food intake, the SPT (sucrose preference test, SPT) was not included in this acute stress protocol to avoid potential confounds.

OVX rats showed a significant increase in immobility time in FST (Figure 3B). In the OFT experiment, no difference was shown in OFT distance (Figure 3D); however, we recorded decreased OFT central time in OVX rats (Figure 3C). We also observed OFT central time improvement in E2 supplement rats, but no significance. We also evaluated the serum E2 level (Figure 3F). As above, OVX rats showed heavier body weight than other groups (Figure 3E), which means E2 deficiency was closely related to metabolism. We then performed untargeted metabolomics analysis.

### 3.4. ERT Reduces Neuronal Injury in Ovariectomized Rats

**Figure 4:**
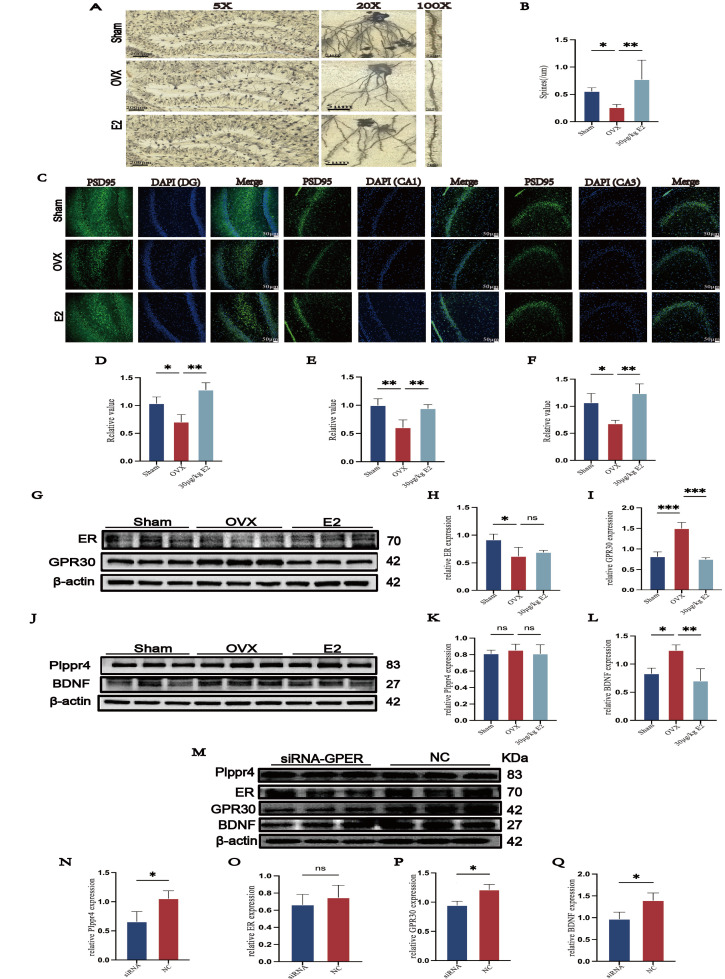
ERT reduces neuronal injury in ovariectomized rats. (**A**) Golgi staining showed dendritic spine density in sham, OVX, and ERT groups of rats. (**B**) Statistical analysis of dendritic spine density. (**C**) Fluorescence intensity of PSD95 (green) in subregions of hippocampus (DG, CA1, CA3). (**D**–**F**) Statistical analysis of PSD95 fluorescence intensity. (**G**) Representative Western blots of ER and GPR30 in the hippocampus. (**H**,**I**) Analysis of hippocampal ER and GPR30 expression in all groups. (**J**) Representative Western blots of Plppr4 and BDNF in the hippocampus. (**K**,**L**) Analysis of hippocampal Plppr4 and BDNF expression in all groups. (**M**) Representative Western blots of Plppr4, ER, GPR30, and BDNF in the SH-SY5Y cell line of the siRNA and NC groups. (**N**–**Q**) Analysis of Plppr4, ER, GPR30, and BDNF expression in the siRNA and NC groups. Statistical analysis was performed using one-way ANOVA with Tukey’s multiple comparisons test. * *p* < 0.05, ** *p* < 0.01, *** *p* < 0.001, ns, no significant difference vs. OVX group.

The short-term effects of E2 on the neurons in ovariectomized rats were further evaluated. Analysis of Golgi-stained samples indicated a significant decrease in hippocampal dendritic spine density following OVX compared with sham-operated controls. This decrease was reversed by ERT, consistent with a protective role of estrogen in spine maintenance (Figure 4A). The analysis results showed a statistically significant difference (Figure 4B). Immunofluorescence was used to detect synaptic function. The results revealed a reduction in PSD95 fluorescence density in hippocampal subregions, including DG (Figure 4C,D), CA1 (Figure 4C,E), and CA3 (Figure 4C,F). ERT increased PSD95 fluorescence density in ovariectomized rats.

Then, the expression of the estrogen receptor was examined. Compared with the sham group, the OVX group presented decreased ER expression (Figure 4G,H), and expression of GPR30 was also elevated (Figure 4G,I); these changes were reversed by ERT. Subsequently, we examined Plppr4 and BDNF expression by WB, and expression of ER and GPR30 was also evaluated. The result showed OVX caused an increase in BDNF expression (Figure 4K,M), and no difference was observed in Plppr4 expression (Figure 4K,L). Consequently, we used siRNA to silence GPR30 in SH-SY5Y. Our results showed that the expression of BDNF reduced significantly (Figure 4M,N), while the expression of Plppr4 (Figure 4M,O) and ER (Figure 4M,Q) did not have the same trend. According to the result, we hypothesized that it was GPR30, rather than ER, that was involved in mediating the neuronal response to short-term stress and that E2 alleviated stress-induced neuronal injury.

### 3.5. ERT Relieves an Abnormal Reduction in Microglia and Inflammatory Proteins in the Hippocampus of OVX Rats

**Figure 5:**
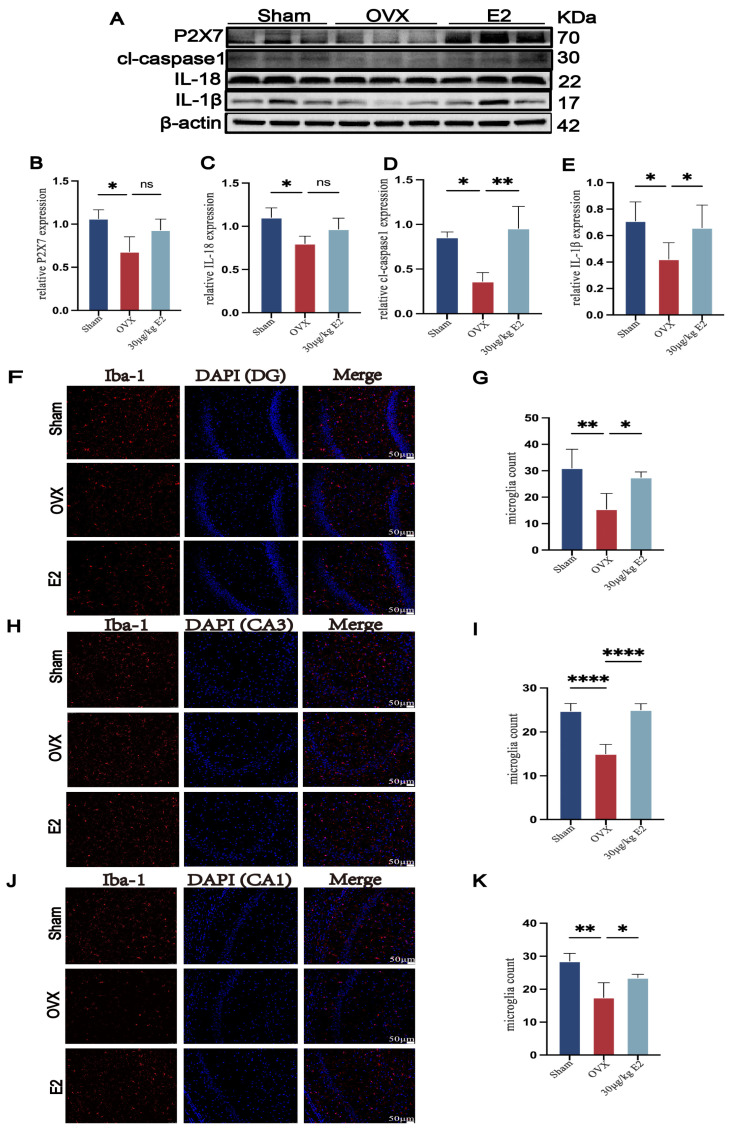
ERT relieves an abnormal reduction in microglia and inflammatory proteins in the hippocampus of OVX rats. (**A**) Representative Western blots of P2X7, IL-18, cl-caspase1, and IL-1β in the hippocampus. (**B**–**E**) Analysis of hippocampal P2X7, IL-18, cl-caspase1, and IL-1beta expression in all groups. (**F**,**H**,**J**) Fluorescence microglia count of Iba-1 (red) in subregions of the hippocampus (DG, CA1, CA3). (**G**,**I**,**K**) Statistical analysis of Iba-1 fluorescence microglia count. Statistical analysis was performed using one-way ANOVA with Tukey’s multiple comparisons test. * *p* < 0.05, ** *p* < 0.01, **** *p* < 0.0001, ns, no significant difference vs. OVX group.

To investigate the role of E2 in regulating the microglial response and neuroinflammation following the depressive-like behaviors in rats, we first examined the expression of P2X7, IL-18, cl-caspase1, and IL-1β. Surprisingly, after OVX, rats showed lower inflammation levels in the hippocampus, and E2 supplement increased expression of P2X7R (Figure 5A,B), IL-18 (Figure 5A,C), cleaved-caspase-1 (cl-caspase-1) (Figure 5A,D), and IL-1β (Figure 5A,E). This pro-inflammatory effect was unexpected based on our previous findings. To identify if microglia were activated, we then used immunofluorescence to detect the number of microglia in hippocampal subregions. Interestingly, we observed a significant IBA-1-positive cell number reduction in OVX rats in DG (Figure 5F,G), CA1 (Figure 5H,I), and CA3 (Figure 5J,K), compared to the sham and ERT groups. We speculate that this abnormal phenomenon could be a result of microglia silence.

### 3.6. ERT Alleviates Neuroinflammation via GPR30/P2X7/NLRP3 Pathway

**Figure 6:**
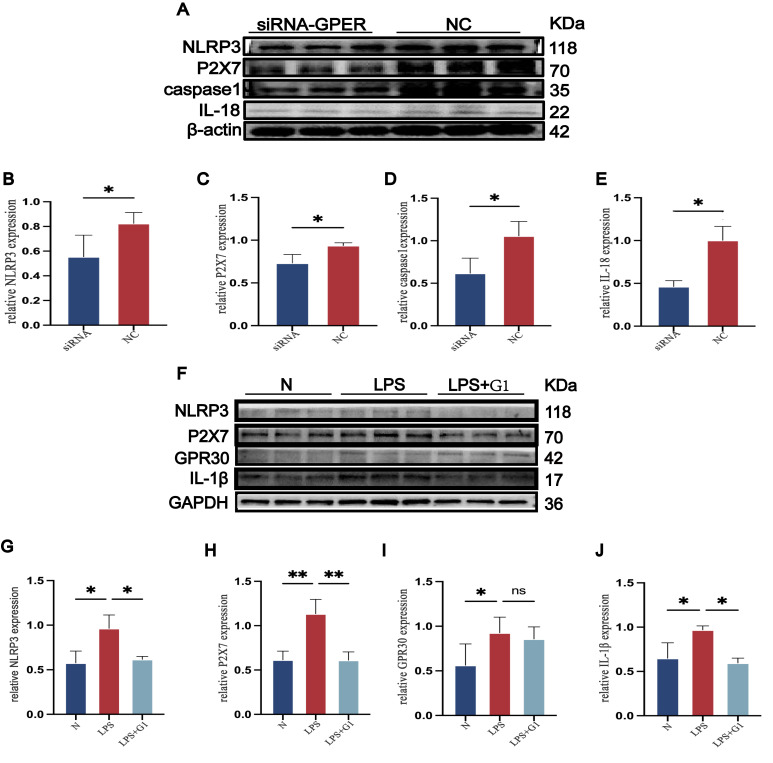
ERT alleviates neuroinflammation via the GPR30/P2X7/NLRP3 pathway. (**A**) Representative Western blot of NLRP3, P2X7, caspase1, and IL-18 in the SH-SY5Y cell line of siRNA and NC groups. (**B**–**E**) Analysis of NLRP3, P2X7, caspase1, and IL-18 expression in siRNA and NC groups. (**F**) Representative Western blot of NLRP3, P2X7, GPR30, and IL-1β in normal, LPS, and G1 groups. (**G**–**J**) Analysis of NLRP3, P2X7, GPR30, and IL-1β in normal, LPS, and G1 groups. Statistical analysis was performed using an unpaired student *t* test (**B**–**E**) or one-way ANOVA with Tukey’s multiple comparisons test vs. the OVX group (**G**–**J**). * *p* < 0.05, ** *p* < 0.01, ns: no significant difference.

A group of siRNAs was used to silence the corresponding GPR30 to identify downstream molecules of GPR30. In SH-SY5Y cells, when GPR30 was silenced (Figure 6A,C), the expression of ER (Figure 6A,B) showed no difference. The expression of NLRP3 (Figure 6F,G), P2X7R (Figure 6F,H), IL-18 (Figure 6F,I), and caspase1 (Figure 6F,J) was suppressed, suggesting that GPR30 is located upstream of these substances. To further confirm this relationship between GPR30 and others, SH-SY5Y cell LPS stimulation was further evaluated. G1 treatment also exhibited a significant anti-inflammatory effect in SH-SY5Y cells. The expression of NLRP3 (Figure 6K,L), P2X7R (Figure 6K,M), and IL-1β (Figure 6K,O) was also elevated in LPS-treated cells, and G1 could inhibit this effect caused by LPS. Another notable finding was that LPS elevated the expression of GPR30; G1 did not present a stronger activation effect (Figure 6F,N), indicating GPR30 was indeed regulated in short-term inflammation. Collectively, these results suggested that E2 could relieve neuroinflammation via the GPR30/P2X7/NLRP3/Caspase1 pathway.

### 3.7. ERT Relieves Serum Metabolic Disorders in Ovariectomized Rats

**Figure 7:**
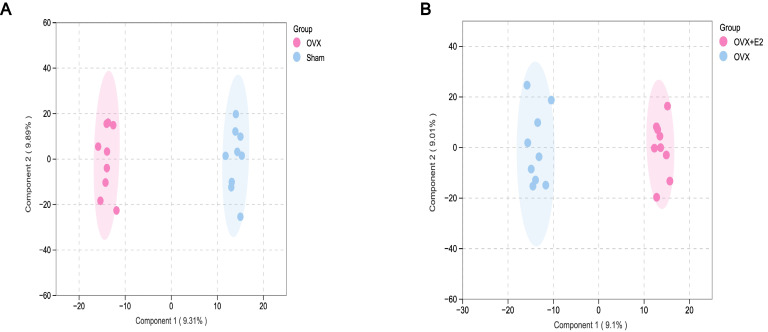

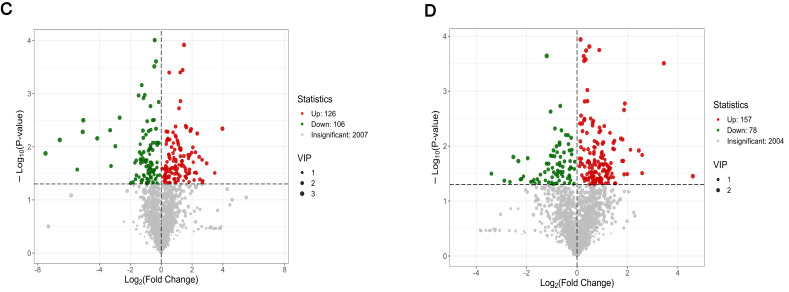

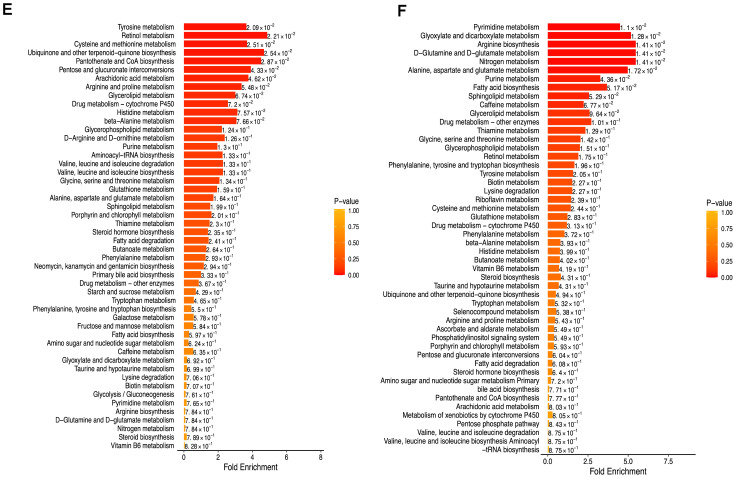
ERT relieves serum metabolic disorders in ovariectomized rats. (**A**) OPLS-DA score plot of the OVX group compared with the sham group. (**B**) OPLS-DA score plot of the OVX group compared with the ERT group. (**C**) Volcano plot of differential metabolites in the sham group and OVX group. (**D**) Volcano plot of differential metabolites in the OVX group and ERT group. (**E**) Pathway enrichment analysis of differential metabolites between the sham group and the OVX group. (**F**) Pathway enrichment analysis of differential metabolites between OVX and E2-treated groups.

To further explore the relationship between E2 deficiency and metabolic disorders, we used LC-MS/MS for untargeted metabolomics analysis of metabolic changes in different samples. We analyzed metabolic profiles of the sham, OVX, and the 30 μg/kg E2 supplement group. The OPLS (Orthogonal Partial Least Squares Discriminant Analysis, OPLS) score plot showed a clear separation between the sham and OVX groups (Figure 7A) as well as between the OVX and E2 supplement groups (Figure 7B). We first compared the metabolomic differences between the sham and OVX groups (Figure 7C) and between the OVX and E2 supplement groups. (Figure 7D) The differential metabolites are illustrated in the volcano plots. Between the sham and OVX groups, we observed that 216 substances were upregulated and 106 substances had decreased expression among 2239 substances (Figure 7C). Between the OVX and E2 supplement groups, we observed 157 substances with increased expression and 78 substances with decreased expression among 2239 substances (Figure 7D). The KEGG enrichment analysis showed that E2 deficiency affected metabolic pathways of sphingolipid metabolism, cysteine and methionine metabolism, and arachidonic acid metabolism, etc. (Figure 7E). Also, E2 supplement affected metabolic pathways of sphingolipid metabolism, arginine biosynthesis, alanine, aspartate, and glutamate metabolism, etc. (Figure 7F). Research has reported that sphingolipid metabolism was related to G-protein receptors and nerve regeneration. We next explore the relationship between GPR30 and the anti-short-term stress effect of E2.

## 4. Discussion

Our findings suggest estrogen deficiency as a key risk factor for depression in women, particularly during the perimenopausal period. Its pathological mechanism involves dysregulation of the GPR30-mediated neuroinflammatory pathway and the P2X7/NLRP3 axis, as well as impaired neuroplasticity. Estrogen replacement therapy may alleviate depressive-like behavior by modulating GPR30 function, inhibiting neuroinflammation, and promoting neural repair.

Compared to male depression, female depression has historically received insufficient research attention, despite women having a twofold higher lifetime risk of developing depression. Epidemiological studies have reported that the incidence of major depressive disorder increases 2- to 5-fold during the perimenopausal period in women [5,25], suggesting a potential link between declining estrogen levels and mood disorders. Nevertheless, the optimal therapeutic strategy for estrogen deficiency depression remains a subject of debate. Although ERT has been proposed as a potential intervention, its effects on mood are complex and appear to be dose-dependent [26,27]. Moreover, studies have yielded conflicting findings regarding the association between systemic ERT use and the risk of depression [28,29].

Based on this background, we first identified and reported the optimal dosage of ERT for alleviating depressive-like behavior in OVX rats. Among the three dosages selected from previous studies [30,31], subcutaneous injection of 30 μg/kg 17β-estradiol (E2) was found to be the most effective. Notably, higher dosages produced differential effects: although 120 μg/kg still conferred behavioral benefits, 60 μg/kg was associated with elevated neuroinflammation compared to the 30 μg/kg dose. To our knowledge, no prior study has systematically examined this dosage issue, and there is currently no consensus regarding the appropriate subcutaneous E2 dosage in OVX rat models. Our study also provides a critical methodological insight: the confounding effect of E2 deficiency on food intake and nutrient absorption suggests that the SPT result in OVX rats should be interpreted with caution, as estrogen deficiency is known to affect metabolic rate and fluid intake, potentially confounding sucrose preference as a measure of anhedonia. Previous animal studies have demonstrated that ERT reduces depressive-like behavior in OVX rats [32]. However, clinical findings remain inconsistent. For instance, a study by Wium-Andersen et al. reported that systemic hormone therapy administered before and during menopause is associated with an increased risk of depression [26]. Similarly, Li et al. found that supraphysiologic doses of 17β-estradiol exacerbate depression-like behaviors in OVX mice [27]. These discrepancies may be partially explained by dose-related effects, as supported by our findings. It has also been suggested that varying hippocampal E2 levels may differentially activate hippocampal subregions. Future studies are warranted to quantify hippocampal E2 concentrations and investigate the region-specific responses of hippocampal subfields to E2 treatment.

Our study further examines the expression of ERs and GPR30 in OVX rats subjected to CUMS. The neuroprotective effects of estrogen are not absolute but appear to be modulated by the expression levels of its receptors. Supporting this, Ma et al. demonstrated that ERT exerts protective effects on hippocampal neurons in OVX mice. In line with this, our results revealed a significant upregulation of ER expression following CUMS and estrogen deficiency. To further assess neuronal injury and neuroinflammation induced by CUMS and estrogen depletion, we measured the expression of BDNF and inflammatory cytokines, as previously described [33]. Based on our observations, we propose that OVX rats may upregulate ER expression in response to prolonged stress as a compensatory mechanism to sustain neurotrophic support and modulate neuroinflammation. In contrast, GPR30 does not appear to play a similar role under these conditions. Notably, the protective effect of ERT diminished under conditions of long-term estrogen deprivation, which may be attributed to reduced ER expression in the hippocampus [34]. Consistent with this, another study reported decreased ERβ expression after four weeks of CUMS exposure [35]. Given that Plppr4 is a postsynaptic membrane-associated protein predominantly expressed in emotion-related brain regions such as the hippocampus and prefrontal cortex [36] and that it interacts closely with LPA2 (Lipoprotein-associated Phospholipase A2, LPA2) [37], a molecule potentially regulated by GPR30 [38], we further investigated whether its expression was altered in our model.

Given that GPR30 has been reported to mediate the rapid, non-genomic actions of estrogen [39], we established a short-term stress animal model involving 10 days of unpredictable stress exposure. Our results showed that OVX rats exhibited increased depressive- and anxiety-like behaviors, confirming successful induction of the stress model. Untargeted metabolomics analysis revealed that E2 treatment alleviated metabolite disturbances associated with sphingolipid metabolism and the biosynthesis of unsaturated fatty acids. Consistent with our findings, previous animal studies—including those in mice and subordinate monkeys—have indicated that sphingolipid metabolism dysfunction is a common feature in mammalian models of depression [40,41]. Moreover, a causal relationship has been suggested between lipid metabolism disorders (e.g., downregulation of long-chain fatty acids and upregulation of phospholipids) and the pathophysiology of depression. Additional metabolic pathways of interest include those linked to HPA axis regulation, such as arachidonic acid metabolism and arginine biosynthesis [42,43]. The HPA axis plays a central role in the neuroendocrine response to stress, and its overactivation—commonly observed in patients with depression—leads to elevated cortisol secretion, cognitive impairment, and persistent low mood. Based on these findings, we hypothesize that GPR30 may serve as a key mediator of estrogen’s rapid effects in the context of short-term stress exposure.

To further elucidate the underlying mechanisms, we employed an acute stress model to induce neuronal injury. Neuronal loss is a critical pathological factor in depression, particularly in brain regions such as the cortex, limbic system, and hippocampus. Chronic stress is known to cause neuronal atrophy or permanent loss, thereby contributing to the development of depressive behaviors [43,44]. Our results demonstrated that even 10 days of stress exposure was sufficient to induce significant neuronal and synaptic damage.

Given that GPR30 mediates rapid, non-genomic estrogen signaling [39] and that the GPR30-selective agonist G1 has been shown to mimic the enhancing effects of 17β-estradiol on hippocampal memory independently of classical estrogen receptors (ERα/ERβ) [45], we investigated the role of GPR30 in neuroprotection. Using siRNA to knock down GPR30 in SH-SY5Y human neuronal cells, we found that GPR30 contributes to neuroprotection, consistent with previous reports implicating the PI3K/AKT pathway [46] and rapid BDNF release [47] in GPR30-mediated effects. Our novel finding is that GPR30 upregulates Plppr4 and BDNF in response to stress, thereby promoting neuroplasticity and neurogenesis to repair acute stress-induced neuronal injury.

Neuroinflammation is a core feature of depression, characterized by aberrant immune responses in the brain and closely linked to microglial activation, which impairs synaptic function and neural plasticity [48,49]. In contrast to the chronic stress model, the short-term stress protocol yielded an unexpected pattern: OVX rats exhibited lower hippocampal expression of P2X7, IL-1β, IL-18, and cleaved caspase-1, along with a reduced number of Iba-1-positive cells. This finding is opposite to the pro-inflammatory effect of estrogen deficiency observed under chronic stress and differs from some previous reports [50]. Several explanations may account for this discrepancy. First, acute or chronic stress may engage different temporal phases of neuroimmune adaptation, with an early hypo-responsive state followed by later hyperactivation. Second, estrogen deficiency could alter the threshold or set point of microglial responsiveness to acute stressors, leading to blunted rather than enhanced inflammatory signaling. Third, the disturbances in sphingolipid metabolism detected by our metabolomics analysis might influence inflammasome priming or microglial survival. We reported that short-term stress combined with estrogen deficiency leads to reduced Iba-1 immunoreactivity and downregulation of P2X7/NLRP3-related proteins in the rat hippocampus. Whether this reflects a genuine functional hypo-response, a loss of microglial cells, or simply reduced expression of the Iba-1 marker warrants further investigation using complementary methods such as flow cytometry, Iba-1/NeuN double staining, or assessment of microglial morphology. Our findings suggest that under intense stimulation, such as systemic stress, activated GPR30 signaling engages anti-inflammatory programs to protect neurons from severe damage.

The P2X7/NLRP3 pathway is a central inflammatory signaling axis in innate immunity, driving inflammatory responses through the P2X7R/NLRP3/caspase-1/IL-1β cascade. To confirm the anti-inflammatory role of GPR30, we used siRNA to knock down GPR30 in SH-SY5Y cells and treated them with LPS alone or in combination with the GPR30 agonist G1. In SH-SY5Y human neuroblastoma cells, knockdown of GPR30 reduced the expression of NLRP3, P2X7, caspase-1, and IL-18, while treatment with the GPR30 agonist G1 attenuated LPS-induced upregulation of these proteins (Figure 6). These in vitro findings suggest that GPR30 can modulate components of the P2X7R/NLRP3 pathway in a neuronal cell line. However, whether this regulation occurs in specific neuronal populations or involves glial cells in vivo remains to be determined. The P2X7 receptor, a homotrimeric ATP-gated cation channel [51], senses extracellular ATP levels via its extracellular domains, which regulate channel opening and closing [52]. Upon ATP activation, P2X7 induces K^+^ efflux, triggering NLRP3 inflammasome assembly and subsequent caspase-1 activation, leading to maturation and secretion of IL-1β and IL-18. In addition, P2X7 is involved in regulating cellular immune functions, such as assisting macrophage-mediated pathogen clearance [53].

Notably, we observed that co-treatment with G1 and LPS also downregulated the P2X7R/NLRP3 pathway. The effects of GPR30 activation appear to be context-dependent. For example, GPR30 activation has been shown to inhibit pro-inflammatory M1 microglial polarization and promote anti-inflammatory M2 polarization via the ERK-NF-κB pathway, thereby alleviating neuroinflammation [54]. Conversely, in neural injury models, GPR30 expression and function are upregulated in DRG (dorsal root ganglion, DRG) neurons and microglia, leading to neuronal sensitization and exacerbated neuroinflammation [55]. Our results indicate that, in this cell model, GPR30 can modulate the P2X7R/NLRP3/caspase-1 pathway. In the short-term stress animal model, ERT reversed the reduction in Iba-1-positive cells and inflammatory proteins observed in OVX rats. Taken together, these findings suggest a potential link between estrogen signaling via GPR30 and the regulation of neuroinflammatory pathways, but the causal relationship and cell-type specificity in vivo require further investigation.

This study has several limitations. First, all experiments were conducted in animal models, and further validation is required to assess the translational relevance of our findings. Second, as we focused on GPR30, we only measured the total expression of ERs; the specific contributions of ERα and ERβ to depressive-like behavior have not been fully evaluated. Nevertheless, the observation that ERT reversed depressive-like behavior and neuroinflammation caused by estrogen deficiency suggests that estrogen depletion itself is a major driver of the observed phenotypes. Third, during our whole experiment, we did not observe any difference in RNA because of time and technology deficiency; we will manage to further explore the RNA mechanism in our next experiment. All mechanistic inferences from SH-SY5Y cells require validation in primary neurons or in vivo models with cell-type-specific manipulations. And although the Sham group was included to validate the OVX model, this study was primarily designed to compare OVX and ERT groups under different stress conditions. Therefore, not all possible pairwise comparisons were performed. In the CUMS dose–response experiment, each group began with 6 rats, but animal deaths during the 28-day protocol reduced the final sample size to 5 per group. This limits the statistical power for detecting effects of higher E2 doses; therefore, these findings are preliminary, and the efficacy of 30 μg/kg was confirmed in a separate cohort (*n* = 9). Future studies with larger sample sizes and more comprehensive group designs are warranted to further explore the differential contributions of ER subtypes and GPR30 under physiological and pathological conditions.

## 5. Conclusions

Estrogen deficiency can lead to depressive-like behavior in female rats, accompanied by neuroinflammatory activation and decreased neuroplasticity, as well as metabolic pathway abnormalities. ERT can reverse these changes. There is a dose–response relationship between ERT and depression-like behavior improvement, and the optimal therapeutic dose is 30 μg/kg.

In acute and chronic stress models, compared with the sham group, OVX group rats showed significant differences in GPR30 and ER, indicating that estrogen mediates acute and chronic depression-like behavior through GPR30 and ER, respectively. In the acute stress model, the neuroplasticity of depressed rats is reduced, and inflammation levels decrease due to abnormal silencing of microglia. In addition, in the acute stress model, GPR30 mediates the occurrence and development of depression through the P2X7/Plppr4 and P2X7/NLRP3/caspase1 pathways. This study reveals the synergistic effect of ER and GPR30 in estrogen deficiency-related depression.

## Data Availability

The raw data supporting the conclusions of this article will be made available by the authors on request.
